# Pathophysiology of SARS-CoV-2-associated ischemic stroke is variegated

**DOI:** 10.1186/s41983-021-00378-1

**Published:** 2021-09-08

**Authors:** Fulvio A. Scorza, Ana C. Fiorini, Josef Finsterer

**Affiliations:** 1 Disciplina de Neurociência, Universidade Federal de São Paulo/Escola Paulista de Medicina (UNIFESP/EPM), Rua Pedro de Toledo, 697 - Vila Clementino, São Paulo, 04039-00 SP Brasil; 2 Programa de Estudos Pós-Graduado em Fonoaudiologia, Pontifícia Universidade Católica de São Paulo (PUC-SP), São Paulo, Brazil; 3 Departamento de Fonoaudiologia, Escola Paulista de Medicina/Universidade Federal de São Paulo (EPM/UNIFESP), R. Botucatu, 740 - Vila Clementino, São Paulo, SP 04023-062 Brazil; 4 Klinik Landstrasse, Messerli Institute, Postfach 20, 1180 Vienna, Austria

**Keywords:** Ischemic stroke, SARS-CoV-2, COVID-19, Pathophysiology, Cardiac

With interest we read the review article by Roushdy et al. about the pathophysiological mechanisms involved in the development of SARS-CoV-2-associated stroke [1]. SARS-CoV-2-associated stroke mechanisms discussed were affection of the renin angiotensin system and angiotensin-converting enzyme 2 (ACE-2) receptor downregulation, endothelial cell damage with coagulopathy, cytokine storm, and platelet dysfunction [1]. The review is appealing but raises the following comments and concerns.

The spectrum of pathophysiological mechanisms of stroke occurring in patients with SARS-CoV-2 is wider than anticipated. Since the heart can be primarily or secondarily affected in COVID-19 patients [2], cardiovascular causes of SARS-CoV-2-associated stroke should be considered. Cardiac manifestations of COVID-19 include myocarditis, coronary heart disease and myocardial infarction, arrhythmias, Takotsubo cardiomyopathy, and heart failure [2]. If any of these conditions is complicated by thrombus formation within the left ventricle, such as in myocarditis, heart failure, Takotsubo cardiomyopathy, myocardial infarction, or arrhythmias, cardio-embolic stroke may ensue.

Thrombo-embolic ischemic stroke may also result from deep venous thrombosis and presence of a patent foramen ovale (PFO). Since thrombosis is a common complication of COVID-19 [3], patients with a PFO are at increased risk of experiencing an embolic stroke, unless they are sufficiently anticoagulated in due time.

Cardiac involvement in COVID-19 may also include affection of the autonomic fibres if COVID-19 is complicated by Guillain–Barre syndrome (GBS), which can affect not only motor and sensory fibers but also autonomic fibers. GBS is increasingly recognised as a neurologic complication of COVID-19 [4]. Affection of the autonomic innervation of the heart may be complicated by Takotsubo cardiomyopathy or arrhythmias and thus ischemic stroke.

A further pathophysiological mechanism of ischemic stroke in COVID-19 patients is vasospasm of cerebral arteries. Arterial vasospasms complicated by ischemic stroke may not only occur after subarachnoid bleeding (SAB), posterior reversible encephalopathy syndrome (PRES), or migraine but also in SARS-CoV-2-associated encephalopathy [5]. None of the COVID-19 patients with cerebral vasoconstriction syndrome experienced SAB, PRES, or migraine.

Coagulopathy leading to ischemic stroke may not only derive from activation of plasmatic coagulation factors by the virus or the reaction to it but also from activation of the coagulation system in the context of sepsis. Sepsis is a common complication of COVID-19 as the virus may weaken the cellular and humoral immune system and thus may give rise to bacterial, viral, or fungal superinfections [6].

It is unclear if “stroke” means ischemic stroke or if the term is used for covering ischemic stroke, intracerebral bleeding, and SAB. A clear delineation and definition of the term is crucial as pathophysiology of the stroke subtypes may vary considerably between each other.

In conclusion, the pathophysiological background of SARS-CoV-2-associated stroke is broader than anticipated. Considering all potential pathophysiological mechanisms of SARS-CoV-2-associated ischemic stroke is crucial, as it may determine the therapeutic management and thus the outcome of patients with COVID-19 complicated by ischemic stroke.

## Data Availability

All data reported are available from the corresponding author.

## Abbreviations

ACE-2: Angiotensin-converting enzyme 2
GBS: Guillain–Barre syndrome
PFO: Patent foramen ovale
PRES: Posterior reversible encephalopathy syndrome
SAB: Subarachnoid bleeding
